# Caring in Context: Development of a Family-Centred and Cross-Sectoral Framework to Support Young Carers

**DOI:** 10.3390/healthcare14060712

**Published:** 2026-03-11

**Authors:** Marianne Frech, Martin Nagl-Cupal, Steffen Kaiser, Anna-Maria Spittel

**Affiliations:** 1 Solothurner Spitäler AG, 4500 Solothurn, Switzerland; marianne.frech@spital.so.ch; 2 Department of Nursing Science, University of Vienna, 1080 Vienna, Austria; martin.nagl-cupal@univie.ac.at; 3 Department of Education of People with Intellectual and Developmental Disabilities, Institute for Special Education, Europa-Universität Flensburg, 24943 Flensburg, Germany; steffen.kaiser@uni-flensburg.de; 4 Department of Special Needs Education and Rehabilitation, Special Needs Education, Rehabilitation and Health Care, Carl von Ossietzky Universität Oldenburg, 26129 Oldenburg, Germany

**Keywords:** framework, support, Switzerland, young adult carer, young carer

## Abstract

**Background/Objectives:** Children and adolescents who care for family members with illness, disability, or mental health conditions face challenges across educational, health, and psychosocial domains. Although research and practice have developed conceptual models and assessment tools to better understand and address young carers’ situations, a persistent gap remains between their needs and available support, reflecting structural fragmentation across health, education, and social care systems. To address this gap, this article presents the development of a family-centred framework spanning these sectors. **Methods:** The framework was developed through an iterative, empirically grounded process based on two studies within a larger research project on young carers in Switzerland. Key themes, structural challenges, and support-related factors were identified by systematically synthesising the findings of the two studies and integrated into an overarching framework linking young carers’ family contexts with cross-sectoral service structures. **Results:** The *Caring in Context Framework* synthesises empirical findings into a coherent framework for understanding and addressing young carers’ situations. By systematically extending the whole family approach to include a cross-sectoral dimension, it bridges relational family dynamics and structural service contexts. Sustainable support is conceptualised as dependent on the structural visibility and institutional recognition of young carers across all system levels, positioning identification and recognition as prerequisites for coordinated responses in research, policy, and practice. **Conclusion:** The framework advances conceptual clarity by integrating family-centred and cross-sectoral perspectives. Rather than creating new services, it emphasises adapting and coordinating existing structures while ensuring systematic recognition of young carers to support coherent, sustainable, and inclusive strategies.

## 1. Introduction: Young Carers and Their Need for Support

Throughout the often volatile course of illness, impairment, and frailty, individuals and families face various challenges. They try to cope with new and demanding life situations through support from family members or the involvement of professional services. These collaborations of care across different care settings are complex [1,2]. Informal caregiving in health and social contexts is predominantly carried out by adults. In many European countries, efforts to curb rising health expenditures increasingly rely on the active involvement of citizens in family care arrangements [3]. This engagement is increasingly recognised within both research and policy discourses, alongside the recognition that assuming a caregiving role is often less a matter of choice than a structural necessity when a family member requires care [4,5].

While informal caregiving is predominantly associated with adults, growing attention has been directed toward children, adolescents, and young adults who also assume caregiving roles within their families. Their contributions—often hidden, unacknowledged, or underestimated—add an additional layer of complexity to informal care arrangements [6,7,8,9,10]. Young carers may take on practical, emotional, or supervisory tasks, frequently navigating responsibilities that shape their development, educational opportunities, social participation, and overall well-being. Understanding their specific needs and vulnerabilities is therefore essential when examining informal caregiving across the lifespan [11,12,13,14,15].

As countries reduce social and health services, families are expected to increase their informal support by investing more time, financial means, emotional and physical efforts—including the help and assistance of children and young adults. This redistribution of responsibilities intensifies structural and relational pressure within families living with chronic illness and impairment [16]. Health and social services often fail to adequately address families’ needs for assistance and respite [17,18]. Socio-demographic factors such as family composition, cultural or migration background, low income or poverty, educational level, gender, and family care preferences influence how families cope with supporting someone close in need of support and care [19,20]. When care provision focuses primarily on the individual with illness or impairment rather than on the family system as a whole, and when appropriate support services or information are lacking, children and adolescents may assume care responsibilities to stabilise family life in challenging circumstances [21,22]. Within families, caregiving responsibility is often taken on at a very early age, including during childhood; however, this level of personal involvement is frequently not age-appropriate, and the care provided by young people remains largely unrecognised, both within the family context and by professionals in the health, social, and educational sectors [23].

These young carers—children and adolescents who take on substantial caring responsibilities for family members living with illness, disability, or mental health conditions—have increasingly been recognised within health, education, and research. Previous studies have documented elevated risks related to physical and mental health, educational participation, and social inclusion [24].

By definition, young carers are under the age of 18 years and look regularly after a person who is affected by illness or impairment. They provide or intend to provide care, assistance, and support for the person in need [6,25]. Young people between 18 and 24 years who provide informal care are referred to as “young adult carers” [26]. From the perspective of family members or close others living with illness, impairment, or mental health conditions, these children and young people often assume adult-like responsibilities under stressful conditions, creating a precarious balance between caregiving demands and other important areas of life, such as education and social participation [25,27]. This is particularly noteworthy as young carers are significant in numbers: across Europe, prevalence rates of young carers vary between 3.5% up to 8% [9,28]. In Switzerland, the prevalence among young carers aged 10 to 15 years is close to 8% [8], suggesting that one or two young carers may be present in every classroom. Despite these numbers, limited public and professional awareness leaves these young carers alone with their challenges and increases the negative impacts of their caring role on their further development [10,29,30].

The impact of caregiving on children and young adults is well documented and underscores their heightened vulnerability [13,31,32,33,34,35]. Research consistently demonstrates that young carers experience interrelated physical, emotional, social, and educational strains rather than isolated difficulties. Commonly reported adverse outcomes include persistent worries, sleep disturbances, and limited opportunities for respite, all of which can adversely affect mental health [11,14,15,27,32]. In particular, when caring for family members with stigmatised conditions such as mental illness, addiction, or illnesses with negative societal perceptions, young carers frequently encounter misunderstanding and social stigma [13,36]. This stigma often reinforces secrecy within the family and restricts access to external support [37]. Such cumulative burdens shape their transition into adulthood, influencing autonomy, social relationships, and educational or vocational trajectories [38]. This is particularly consequential given that emerging adulthood represents a pivotal developmental stage during which major personal and professional decisions are made [39].

The international research discourse over the last three decades has built a growing evidence base and a deeper understanding of young carers and young adult carers within national contexts. A reliable in-country research database is considered one of the key factors for improving the situation of young carers and for raising awareness amongst professionals and the public [9,28,40]. Consequently, international research also implies that a nationwide advocacy group for carers may play a central role in (a) identifying young carers and (b) raising public awareness of their situation [9,23,28].

Institutionally, adopting a family-centred approach emphasises the necessity of interdisciplinary collaboration between healthcare, education, and social services to holistically address young carers’ multifaceted needs [41]. Such an approach encourages flexible educational structures and tailored support initiatives, including comprehensive teacher training and recognition systems like the “Young Carers in Schools” initiative, which actively involves young carers in shaping the services they receive [40,41,42]. A genuinely interdisciplinary, family-centred approach addresses the needs of the family as a whole and integrates informal and formal support across health, education, and social care systems. By embedding such an approach, institutions can better accommodate young carers’ dual roles, ensuring equitable opportunities for educational success and personal development without compromising essential caregiving responsibilities. Support for young carers is strongly shaped by community- and system-level conditions. Studies show that interventions such as preventive education, psychosocial programmes, peer support, and family communication exist, but are often implemented ad hoc and lack coherent conceptual anchoring and sustained cross-sectoral coordination [43]. Young carers emphasise the need for recognition of their role, age-appropriate information, and coordinated professional and peer support: needs that remain insufficiently met in fragmented service landscapes. At the same time, evidence shows elevated risks for health and educational outcomes, while highlighting that supportive contexts can foster resilience, coping, and benefit-finding, particularly through recognition, emotional support, and peer-based approaches [31,44,45,46]. These findings point to a persistent structural gap between young carers’ needs and existing support arrangements.

The relevance of these structural gaps extends beyond national contexts and aligns with international policy frameworks. The United Nations Sustainable Development Goals (SDGs) provide a global agenda emphasising equitable access to health, education, and social participation. This is particularly relevant in light of SDG 3 (promotion of mental health and well-being); SDG 4 (elimination of disparities in education); and SDG 10 (Reduced Inequalities), which aims to reduce social and health disparities within and among countries [47]. Young carers’ heightened risks regarding mental health, educational participation, and social inclusion directly intersect with these targets. Addressing their needs through coordinated, family-centred and cross-sectoral strategies therefore contributes not only to national welfare objectives but also to internationally agreed development goals.

To conceptualise and respond to young carers’ situations, research and practice have developed a range of models and assessment tools over recent decades. The *Tripartite Model of Support* [48] has been developed to understand and investigate the elements of youth caregiving with regard to parental illness by focusing on responsibilities, experiences, and tasks. Widely used validated instruments include the *Multidimensional Assessment of Caring Activities Checklist MACA-YC18* and *the Positive and Negative Outcomes of Caring Questionnaire (PANOC-YC20)* [40]. Metzing [49] developed a model referring to the experiences of young people with caring responsibilities and the construction of familial care. This model can be used to understand influencing factors as well as resources and burden of the caring role. A more practice-oriented approach is reflected in the *Young Carer Service Model* [50], which emphasises structured assessments and individualised goal setting with young carers. Lewis’ model emphasises that support becomes effective only once young carers are socially identified and recognised by services. Rather than focusing on tasks or deficits, the model highlights identification, validation, and relational support as key entry points, with services playing a crucial role in transforming hidden family responsibilities into an acknowledged social role. In this way, services are not merely providers of help but active agents in shaping young carers’ access to support and their developing identities. The model presented by Nagl-Cupal et al. [51] is a concise logic model that structures the essential components required for effective and sustainable support for young carers and their families. It delineates the contextual preconditions, such as public and professional awareness and the ability to identify young carers, as foundational factors shaping access to support, and outlines the material, human, organisational, and societal resources necessary to operationalise interventions. Based on these prerequisites, the model specifies measures directed at both young carers and their families, including needs assessment, age-appropriate information, counselling, opportunities for respite and peer exchange, as well as family-oriented guidance and coordination. It further anticipates short- and long-term outcomes at individual, family, and societal levels and embeds continuous evaluation as an integral component to ensure quality, coherence, and sustainability of support across sectors.

Despite these conceptual and practical advances, a persistent gap remains between young carers’ needs and available support, reflecting broader tensions within health, education, and social systems. Fragmented national structures, limited intersectoral coordination, and low professional awareness contribute to inconsistent recognition and limited access to assistance [30]. Families affected by chronic illness or impairment report that services prioritise the needs of the ill person while overlooking the experiences and needs of children and young people involved in care [17]. Consequently, many young carers continue to assume responsibilities beyond their age and developmental capacity, often without recognition or guidance by professionals or by the families affected.

In order to address the systemic gap between young carers’ needs and available support, this article develops and applies an integrative, family-centred, cross-sectoral theoretical framework to examine how professional responses, family support dynamics, and collaboration across health, social, and educational sectors shape timely access to appropriate support.

It asks what factors, structures, and professional practices are required to ensure effective and sustainable support for young carers and their families within fragmented care systems. By conceptualising support as a relational, family-centred, and cross-sector responsibility, the article advances an integrative perspective on strengthening professional recognition, coordination, and collaboration to prevent unmet and age-inappropriate care arrangements. This integrative perspective underpins the development of a theoretical framework as a practice- and policy-oriented response to these challenges. While existing models have significantly advanced the conceptual and practical understanding of young carers’ situations, they do not consistently articulate how family-centred approaches can be systematically embedded within cross-sectoral service structures.

We present and discuss a theoretical framework developed as an emergent and integrative outcome of a larger research project on young carers. In addition to its empirical grounding in the Swiss research context, the Caring in Context Framework has been applied and further refined in collaborative exchange within the German-speaking region (Germany, Austria, and Switzerland). Members of the interdisciplinary author team have engaged in practice-oriented implementation, academic dialogue, and professional training activities across these contexts, allowing iterative adaptation of the framework to diverse institutional settings. This cross-national application has contributed to sharpening its coordination function and enhancing its practical transferability beyond the original research setting.

Building on the whole family approach to include cross-sectoral dimensions, the framework highlights how the familial context and care responsibilities of young carers intersect with structural service contexts and offers practical guidance for research, policy, and practice. Therefore, it provides a novel lens to understand young carers’ needs, emphasises the importance of professional recognition across sectors, and guides the development of coordinated, family-centred support strategies.

## 2. Materials and Methods

The underlying research project was conceived as a doctoral dissertation at the University of Vienna [52] embedded within the broader Swiss National Science Foundation (SNSF) project *Young Carers and Young Adult Carers in Switzerland* (Project No. 160355), conducted between 2015 and 2020. The national project aimed to establish a comprehensive evidence base on the situation, needs, and support structures of young carers and young adult carers by integrating conceptual, quantitative, and qualitative approaches. Within this larger research framework, the dissertation pursued the overarching question of what constitutes effective and sustainable support for young carers and their families. It thereby contributed an in-depth analysis designed to inform the development of a coherent, cross-sector and family-centred framework for practice and policy [52].

To achieve these aims, namely, to identify key factors for effective and sustainable support for young carers and their families, this article draws on two interrelated empirical studies, which together were designed to capture professional perspectives across institutional contexts as well as the lived experiences of young carers and their families. The framework was created through an iterative and empirically grounded process, drawing on two previously published studies that examined the situation of young carers and their families [53], as well as professional recognition and support [30,54]. Findings from these studies were systematically reviewed and synthesised to identify overarching themes, structural challenges, and support-related factors, which were then integrated into a conceptual framework linking the familial situation of young carers with cross-sectoral service structures.

### 2.1. Description of the Data

Study 1: Study 1 focuses on professionals working in education, healthcare, and social services, as these sectors are central to the early identification of young carers and to shaping pathways to support. The primary objective of this quantitative study [30,54] was to examine how young carers and young adult carers are perceived within professional practice, the extent to which they are recognised as a relevant group, and how professionals understand their own responsibilities and capacities in responding to their needs. Nationwide survey data were collected between 2016 and 2018 using a cross-sectional online questionnaire (final analytic sample *N* = 2311 for awareness analyses; *N* = 2142 for support-related analyses). Data were analysed using descriptive statistics (frequencies, percentages, 95% confidence intervals) to describe levels of familiarity, perceived relevance, identification, and support practices across occupational sectors.

To examine sector differences and associations between key awareness indicators (familiarity, relevance, identification, and ability to support), Chi-square tests of independence (χ^2^) were conducted. Where appropriate, effect sizes (Phi, φ) were calculated to estimate the strength of associations. In addition, one-sample z-tests for proportions were applied to test predefined hypotheses regarding awareness levels (<50% threshold). Statistical analyses were performed using IBM SPSS (Version 24/25), with a significance level set at α = 0.05. The analyses identified sector-specific patterns of awareness, differences in attitudes and perceived responsibilities, and structural factors influencing professional responses. These findings provided insight into systemic gaps, uncertainties, and opportunities within existing professional and institutional frameworks.

Study 2: While the quantitative study provides an overview of professional awareness and support provision, it does not capture how support is experienced within families affected by caregiving. To address this gap, a qualitative study [53] was conducted in 2018 involving 20 families with young carers, young adult carers, and care recipients.

Semi-structured interviews were carried out individually or, where preferred, as dyadic interviews. All interviews were audio-recorded, transcribed verbatim, and analysed using qualitative content analysis with both deductive and inductive coding procedures [55]. Coding was conducted iteratively by multiple members of the research team to ensure analytical rigour.

The findings highlight several challenges in professional support. Participants emphasised the importance of receiving age-appropriate information and being included in communication about the illness or impairment. A lack of information often led to uncertainty and emotional strain. Young carers reported developing substantial practical and social competencies, yet many struggled to balance caregiving with school or vocational demands. Educational performance and career planning were sometimes affected. Access to formal support was frequently described as complex and inconsistent. Administrative barriers, unclear responsibilities, and discontinuities in service provision contributed to additional stress. At the same time, positive experiences were associated with professionals who built trustful relationships, acknowledged the situation of the whole family, and provided reliable and continuous support. Opportunities for open conversations—with professionals, teachers, peers, or within the family—were perceived as particularly relieving. Overall, the study underscores the need for coordinated, family-sensitive, and continuous professional support that recognises both the burdens and the capacities of young carers and their families.

Taken together, the two studies provide complementary perspectives on support for young carers: the professional, institutional viewpoint captured through quantitative survey data, and the experiential, family-centred perspective generated through qualitative inquiry. The quantitative analysis identified structural patterns of awareness, recognition, and sectoral differences, while the qualitative study illuminated how these structural conditions are experienced and negotiated within families.

The combination of the two studies enabled an integrated understanding of support needs and support practices, allowing the research to link professional awareness and practices with the lived realities of young carers and their families. The synthesised findings formed the empirical foundation for the subsequent development of an overarching framework for support, grounded in both professional practice and everyday caregiving experiences.

For an overview of methodological characteristics and key findings of both studies, see Table A1 in Appendix A.

### 2.2. Framework Development

Insights from the two studies were iteratively synthesised through an empirically grounded process to inform the development of the framework. The process involved systematically reviewing and comparing findings from both studies in order to identify overarching themes, structural considerations, and aspects of support relevant for young carers and their families. These empirically derived insights were then conceptually integrated with existing theoretical perspectives, namely Tronto and Fisher’s Modes of Care [56] and the Whole Family Approach [57], to create a framework that links relational and moral dimensions of care with systemic and practice-oriented considerations. This iterative, theory-informed synthesis ensured that the resulting framework captures both micro-level dynamics of family care relationships and macro-level structures relevant for institutional and societal recognition, while maintaining a focus on the method of its development rather than reporting specific findings.

All ethical considerations reported refer to the primary studies on which this research project is based. Both studies were conducted in accordance with the principles of the Declaration of Helsinki [58]. Ethical approval for the data collection was granted by the Zurich Cantonal Ethics Committee (Ref. No. 85–2015). Participation in all studies was voluntary. Informed consent was obtained from all participants, and in the case of minors, additional consent was secured from their legal guardians. Throughout all phases of the research process, strict procedures were applied to ensure confidentiality, anonymity, and data protection.

## 3. Results

The following section presents the empirical findings of the quantitative and qualitative studies underpinning this article. To enhance analytical coherence and to avoid a fragmented presentation of study-specific results, the findings are synthesised and systematically integrated into a framework. This approach organises the data across studies and data sources to highlight converging patterns, complementarities, and key factors relevant to support for young carers and their families.

By incorporating perspectives from young carers, care recipients, informal support networks, and professionals across health, education, and social services, this synthesis provides a transparent and empirically grounded basis for this framework as the central outcome of the project.

### 3.1. Key Factors of Support for Young Carers and Their Families

To address the research question concerning the key factors required for effective support for young carers and their families, the following section presents the empirical findings derived from the two studies. The results synthesise evidence from both quantitative and qualitative data, with a focus on patterns, consistencies, and contrasts identified across data sources. For analytic clarity, the findings are organised into three thematic groups reflecting the main stakeholder perspectives examined in the studies: (a) young carers, (b) professionals, and (c) care recipients and family members. This structure reflects how support needs, challenges, and enabling conditions emerged empirically from the data, rather than being predetermined by theoretical assumptions.

**(a)**  **Findings related to Young Carers: Care Burden, Recognition, Information, Daily Demands, Role Appraisal**

Across both studies, young carers emerged as a structurally under-recognised group. The qualitative interviews revealed that many young carers did not self-identify as “young carers”, as caregiving was normalised within the family and rarely explicitly labelled. Caring activities were described as “just part of family life”, which contributed to limited self-recognition and reduced help-seeking. This qualitative finding is mirrored by the quantitative data. In the nationwide survey, only 44.7% (95% CI [42.6–46.9]) of professionals reported being familiar with at least one term describing “young carers”.

Furthermore, only 30.3% perceived the issue as relevant in their occupational context (z = −18.93, *p* < 0.001), and familiarity was significantly associated with identification (χ^2^ (1, *N* = 2311) = 37.744, *p* < 0.001, φ = 0.128).

These findings suggest that limited recognition operates on two levels: within families, where caregiving is normalised and rarely named, and within institutions, where familiarity and structured identification remain inconsistent.

Qualitative findings show that young carers frequently lacked access to clear, age-appropriate information about the illness or impairment of the care recipient. Several reported having to “find out on their own” what a diagnosis meant, which increased uncertainty and emotional strain. Survey data suggest structural conditions underlying these gaps: professionals unfamiliar with young carer terminology were significantly less likely to perceive the issue as relevant (χ^2^ (1, *N* = 2311) = 35.682, *p* < 0.001, φ = 0.124), reducing the likelihood of proactive identification and guidance.

Interview participants also described persistent tension between school, leisure, and caregiving responsibilities, characterised by time pressure, fatigue, and concentration difficulties. Quantitative findings indicate sectoral differences in identification capacity: while 53.1% of professionals overall reported being able to identify young carers, this varied significantly across sectors (χ^2^ (2, *N* = 2311) = 24.326, *p* < 0.001), with education reporting the lowest rates (45.4%) and social services the highest (61.4%). These disparities suggest uneven institutional responsiveness to role conflict.

Caregiving experiences were further shaped by relational dynamics within families. Appreciation and open communication mitigated strain, whereas silence or unclear expectations intensified it. Institutionally, professionals’ ability to support young carers was strongly associated with their ability to identify them (χ^2^ (2, *N* = 2311) = 197.077, *p* < 0.001), highlighting recognition as a key precondition for structured support.

Although young carers described developing independence and organisational skills, these competencies emerged as adaptations to necessity rather than voluntary gains. Despite 55.8% of professionals reporting an ability to support young carers, coordinated and systemic approaches were inconsistently described, indicating that competence development often occurs in the absence of comprehensive support structures.

Across both studies, young carers’ burden appears shaped less by caregiving tasks alone than by structural conditions of limited recognition, fragmented information, and uneven institutional coordination. Limited professional familiarity (44.7%), identification (53.1%), and significant links between awareness and support capacity underscore the systemic nature of these challenges, while qualitative findings illustrate how these gaps are experienced in everyday family life.

**(b)**  **Findings related to Professionals: Awareness and Recognition, Responsibility, Support Practices, Coordination, Training**

Across health, education, and social services, awareness and recognition of young carers remain uneven and structurally fragile. Fewer than half of professionals reported familiarity with relevant terminology (44.7%), and familiarity was significantly associated with both perceived relevance and identification capacity. Identification itself varied across sectors (overall 53.1%), with education reporting the lowest rates (45.4%) and social services the highest (61.4%). These differences indicate that recognition is not systematically embedded but contingent upon sectoral context, training exposure, and individual awareness.

Although 55.8% of professionals reported being able to support young carers, this reported capacity was strongly associated with prior identification, underscoring recognition as a prerequisite for action. Support practices were primarily described as counselling, emotional support, and referral activities. However, these interventions appeared largely informal and situational rather than embedded in shared procedures, standardised pathways, or coordinated intersectoral strategies. Coordination mechanisms were inconsistently described, and collaboration across sectors was often dependent on individual initiative rather than institutional mandate.

Professionals with personal caregiving experience were significantly more likely to report confidence in supporting young carers, suggesting that experiential knowledge enhances sensitivity and responsiveness. At the same time, the absence of structured training and clearly defined responsibilities contributes to practice variability and limits the continuity of support.

Overall, these findings reveal that support for young carers is not primarily constrained by willingness, but by structural conditions: limited awareness, fragmented coordination, and insufficient institutional anchoring. Recognition, therefore, emerges as a foundational mechanism linking awareness, identification, and action. The empirical patterns suggest that effective support requires not only individual competence but systemic embedding across sectors—highlighting the need for a framework that integrates recognition, coordination, and clearly defined responsibilities at multiple levels of practice.

**(c)**  **Findings related to Care Recipients and Family Members: Family Strain, Communication, Role Expectations, Access to Support, System Continuity**

The perspectives of care recipients and family members underscore the systemic interdependence within families affected by illness or impairment. Families described substantial emotional and organisational strain and repeatedly reported that professional services focused primarily on the ill person while overlooking the involvement and needs of children contributing to care. In qualitative interviews, parents emphasised that “the focus is always on the patient,” whereas children’s contributions remained largely unaddressed. This selective focus aligns with quantitative findings showing limited structural recognition: only 44.7% of professionals reported familiarity with young carer terminology, and identification capacity varied significantly across sectors (53.1% overall; χ^2^ (2, *N* = 2311) = 24.326, *p* < 0.001). Limited recognition at the institutional level thus reinforces children’s invisibility within formal care arrangements.

Care recipients expressed marked ambivalence: while they were concerned about the burden placed on their children, they simultaneously depended on their assistance due to financial constraints, limited service availability, or difficulties accessing external support. Survey data indicate that although 57.7% of professionals reported being able to refer young carers to other organisations, referral practices differed significantly by sector (χ^2^ (4, *N* = 2142) = 24.090, *p* < 0.001), suggesting uneven navigation pathways across institutional contexts. Where referral structures were unclear or fragmented, families described having to “manage everything themselves,” leading children to assume compensatory roles.

Communication patterns and illness-related dynamics further shaped caregiving involvement. Interview data revealed that limited openness about illness, stigma—particularly in relation to mental illness or addiction—and unclear role expectations increased uncertainty and anxiety among children. In several accounts, young carers described acting as “the one who keeps things together,” assuming coordinating or mediating roles within the family. These qualitative insights resonate with quantitative evidence showing that professionals’ ability to support young carers was strongly associated with prior identification (χ^2^ (2, *N* = 2311) = 197.077, *p* < 0.001), indicating that where recognition fails, structured engagement is unlikely to occur.

A recurring empirical pattern concerned discontinuity in formal support. Families reported abrupt service interruptions, unclear professional responsibilities, and weak intersectoral collaboration. Although 55.8% of professionals reported being able to support young carers, descriptions of systematic coordination were inconsistent, and collaboration appeared dependent on individual initiative rather than established pathways. The data thus suggest that caregiving intensity within families is shaped less by illness severity alone than by structural conditions of fragmented service provision and limited continuity.

Overall, the combined findings demonstrate that young carers’ responsibilities expand in contexts characterised by low institutional recognition (44.7% familiarity), uneven identification (53.1%), and fragmented referral structures (57.7% referral capacity with significant sector differences). The qualitative evidence illustrates how these structural gaps are experienced within families—as ambivalence, uncertainty, and negotiated responsibility—highlighting continuity of care, coordinated professional involvement, and explicitly family-centred support as central conditions for reducing children’s caregiving burden.

### 3.2. Overall Findings

Overall, the findings across young carers, professionals, and family members reveal a consistent structural pattern: the primary constraints on effective support are systemic rather than individual. Across all perspectives, recognition emerges as the pivotal mechanism linking awareness, identification, and action. Limited professional familiarity and uneven identification capacity illustrate that young carers’ visibility is not consistently embedded within institutional practice. Qualitative accounts demonstrate how this institutional invisibility translates into delayed support, uncertainty, and self-reliance within families. Recognition thus functions as a structural gateway condition rather than merely an attitudinal variable.

A second cross-cutting pattern concerns coordination. Although a substantial proportion of professionals report the ability to support or refer young carers, these practices remain largely informal, sector-dependent, and insufficiently institutionalised. Intersectoral collaboration is inconsistent, and continuity of care is fragile. As a result, families frequently internalise coordination tasks, and young carers assume mediating and organisational roles that extend beyond direct care activities. The data, therefore, indicate that caregiving intensity is shaped not only by illness severity but by the degree of systemic alignment across services.

Third, family-centred engagement emerges as a stabilising or amplifying condition. Where communication is open and professional involvement coordinated, caregiving responsibilities are more clearly defined and less burdensome. Where stigma, unclear expectations, and fragmented services prevail, children’s roles expand to compensate for institutional gaps. Caregiving trajectories are thus co-produced by relational dynamics and institutional arrangements.

Importantly, these mechanisms—recognition, coordination, and family-centred engagement—do not operate in isolation. They interact across interconnected levels: individual experiences, family systems, professional practice, and broader structural conditions. Existing approaches to young carer support tend to address these dimensions separately, focusing either on awareness, specific interventions, or family approaches. The present study contributes to the field by empirically demonstrating their interdependence and by identifying the structural conditions under which young carers’ roles either stabilise or intensify.

By integrating quantitative evidence on institutional response patterns with qualitative insights into lived caregiving realities, the study advances a multi-level understanding of support that shifts the analytical focus from caregiving tasks alone to the broader support ecology in which they are embedded. The findings indicate that professional willingness is insufficient without structural embedding, cross-sector coordination, and clearly defined responsibilities.

The *Caring in Context Framework* is proposed as a response to this empirically identified gap. It translates the integrated findings into a structured, multi-level model that systematically links recognition, coordination, and relational dynamics across sectors and governance levels, embedding young carer support within a comprehensive care ecology rather than treating it as an isolated practice domain.

### 3.3. The Caring in Context Framework

**Figure 1:**
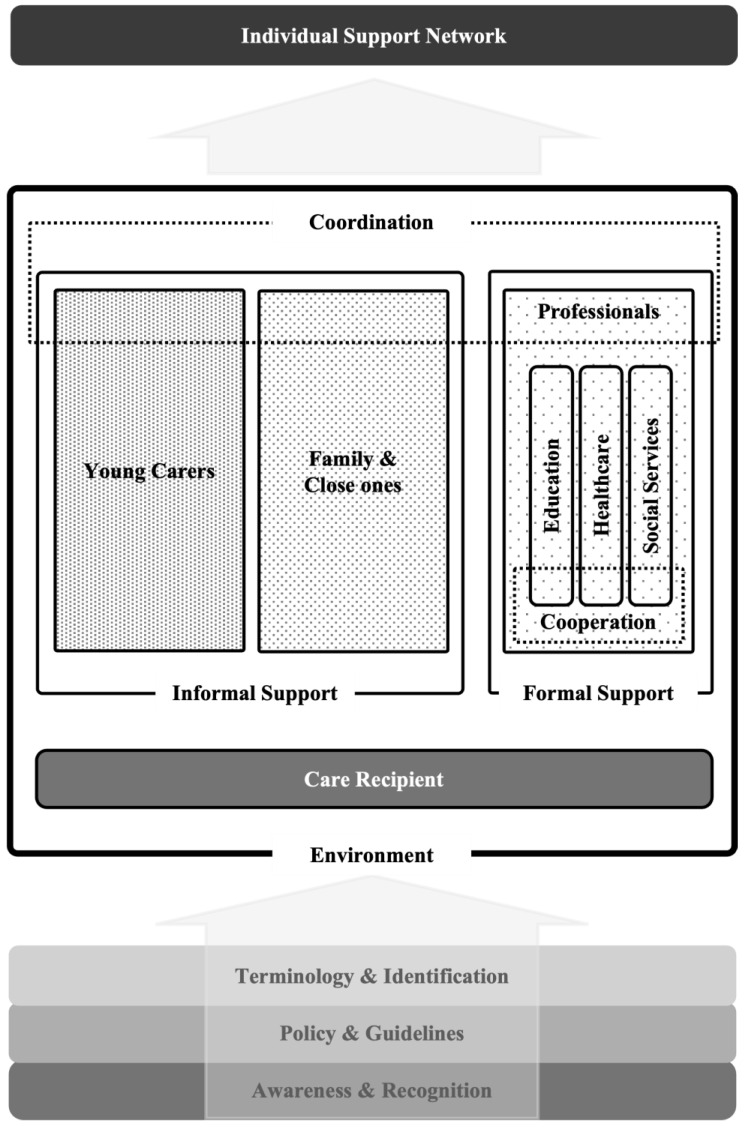
The Caring in Context Framework.

The following section presents the key components of the *Caring in Context Framework* (Figure 1), outlining distinct realms, conditions, and interrelated domains that shape effective recognition, support, and coordination for young carers across individual, family, institutional, and societal realms.

The framework’s underlying foundation is that awareness and recognition, policy and guidelines, and terminology and identification are necessary for successful support of young carers:

**Awareness and Recognition.** Low professional awareness of young carers remains a critical barrier to early identification and support. Consistent with international evidence, many young carers remain “invisible,” preventing timely intervention and resource allocation. To address this, educational curricula at vocational and higher education levels should incorporate modules on the identification of young carers and support strategies, complemented by targeted awareness-raising initiatives for professionals across education, social, and health sectors.

**Policy and Guidelines.** Although national legislation in many countries does not explicitly recognise young carers, their rights are embedded within international human rights frameworks. In particular, the United Nations Convention on the Rights of the Child [59] establishes children’s rights to health (Art. 24), education (Art. 28–29), adequate living conditions (Art. 27), and participation (Art. 12). While a detailed legal analysis is beyond the scope of this article, the Convention provides an essential normative foundation for recognising young carers as rights-bearing individuals within family and care arrangements.

**Terminology and Identification.** A harmonised definition of young carers enhances clarity and facilitates identification. Empirical findings show that families often do not self-identify as having a young carer, limiting access to support services. Therefore, public education and stakeholder engagement campaigns are essential to elevate societal understanding and encourage self-recognition.

This underlying foundation applies to an individual family situation consisting of a defined environment comprising the person who needs support and the informal and formal support systems based on this circumstance:

**Care Recipient Context.** Caregiving roles may evolve gradually in families with chronic conditions or arise suddenly following acute events. In the absence of alternative formal care arrangements—due to limited resources, cultural preferences, or systemic deficits—children assume responsibilities. These dynamics underscore the need for substitute care solutions and resource options at both the family and systemic levels.

**Informal Support.** Informal caregiving develops over time through cumulative life events and dependency relationships. Strengthening informal networks through education, peer support, and community engagement can reduce isolation and more equitably distribute caregiving responsibilities.

**Formal Support and Interprofessional Cooperation.** Effective interventions require professionals to understand family structure, cultural context, and care expectations. Applying established care theories can support respectful, trust-based relationships. A whole-family approach that involves stakeholders from education, healthcare, and social services facilitates seamless coordination of support.

The framework culminates in a coordination phase, wherein practitioners conduct holistic assessments of living environments and support structures to identify unmet needs:

**Coordination.** Collaboratively planning with families and young carers to expand or adapt personal networks enhances coping capacities and coherence. This coordinated, family-centred strategy underpins sustainable support pathways throughout the caregiving trajectory.

**Individual Support Network.** The *Caring in Context Framework* clearly outlines four interconnected realms—*individual, family, institutional,* and *societal*—within which specific processes and conditions determine how support for young carers can be effectively provided and sustained. It draws on the triangulated empirical results and theoretical integration to identify key components influencing recognition, responsibility, and coordination of care:

1. *Individual Realm*: Focuses on personal attributes of young carers, including age, gender, resilience, coping strategies, and their personal experiences of caregiving.
2. *Family Realm*: Examines family dynamics, relationships, and the specific caregiving tasks and responsibilities young carers undertake, influenced by parental illness, disability, or mental health status.
3. *Institutional Realm*: Considers educational and healthcare institutions’ responsiveness, policies, and support structures, assessing their effectiveness and adaptability in addressing the unique needs of young carers.
4. *Societal Realm*: Addresses broader societal attitudes, stigma, policy environments, and the availability of community-based resources, identifying systemic enablers and barriers that affect young carers’ holistic well-being and educational opportunities.

## 4. Discussion

This discussion situates the *Caring in Context Framework*, developed in this research project, within a broader systemic framework that encompasses individual, familial, institutional, and societal dimensions. The framework integrates the empirical findings of the two studies with the theoretical underpinnings of Tronto and Fisher’s Modes of Care [56] and the Whole Family Approach [57,60]. Together, these perspectives position care as a moral, relational, and systemic process and provide a foundation for sustainable, family-centred support for young carers and their families.

Across the theoretical frameworks and empirical findings, awareness emerges as the central structural condition for effective support of young carers and their families. According to other studies and support models [23,50,51], caring responsibilities must first be recognised, named, and legitimised before any form of coordinated or sustainable support can occur. Without awareness, young carers remain structurally invisible within families, professional systems, and policy frameworks, resulting in fragmented or absent support.

### 4.1. Young Carers Support in Recent Research

International research consistently shows that young carers face elevated risks for mental health problems, reduced educational participation, and constrained social inclusion, particularly during key developmental and educational transitions [19,31,33,35]. However, recent studies also emphasise the heterogeneity of caring experiences. While high-intensity caregiving without support is associated with fatigue, distress, and educational disruption, caring under supportive conditions can foster resilience, coping skills, and benefit-finding, especially when young carers experience recognition, emotional support, and opportunities for reflection [44].

Support programmes for young carers on an institutional level are expected to prevent excessive caring roles, mitigate negative impacts, and provide appropriate assistance where caring persists [61].

Persistent unmet support needs among young and young adult carers indicate that existing programmes do not sufficiently integrate preventive measures, mitigation of negative impacts, and appropriate assistance, resulting in continued caring burdens and limited protection against long-term educational, social, and mental health consequences [62].

Community-based research, however, highlights persistent structural limitations in support provision. Although diverse interventions exist (psychosocial programmes, peer support, and family-oriented services), they often lack sustained coordination across education, health, and social care systems [43]. Evaluations of peer-support programmes illustrate both their potential and limitations: they demonstrate positive effects on emotional well-being, social inclusion, and coping, while also revealing challenges related to accessibility, reach, and long-term embedding [45,46].

Taken together, recent research points to three persistent gaps: (1) insufficient integration of education, health, and social care; (2) limited structural anchoring of prevention, transition-sensitive support, and continuity; and (3) a lack of frameworks connecting burden, resources, resilience, and participation. These gaps form the empirical basis for the conceptual contribution of the *Caring in Context Framework*.

### 4.2. Conceptual Added Value of the Caring in Context Framework

Existing models have substantially advanced the understanding of young carers but remain limited in addressing current systemic challenges. Metzing’s [49] grounded theory model conceptualises caregiving as a relational and evolving family process, offering valuable insights into intra-familial dynamics. Lewis’s service-oriented model highlights identification and recognition as critical entry points to support. Meanwhile, the logic model by Nagl-Cupal et al. [51] structures the conditions, resources, measures, and outcomes required for effective support.

The *Caring in Context Framework* extends these approaches by linking individual, family, institutional, and societal levels within a single cross-sectoral framework. It responds directly to empirical evidence showing that fragmented systems, unclear responsibilities, and insufficient professional awareness undermine continuity of support [23,27,43]. By embedding prevention, mitigation, and support across systems, the framework moves beyond isolated interventions toward coordinated care pathways.

Importantly, the framework incorporates insights from resilience and peer-support research without normalising caregiving responsibilities. Positive outcomes are understood as contingent upon recognition, emotional support, professional guidance, and structural embedding rather than individual resilience alone [44,45,46]. In this way, the *Caring in Context Framework* provides a framework that aligns lived experiences, professional practices, and institutional responsibilities:

*Individual-Level Findings.* Outcomes of young people’s caregiving are not uniform; they vary considerably depending on contextual and personal factors, such as age, gender, cultural background, and developmental stage. The data indicate that individual outcomes emerge from the interaction between caregiving demands and the conditions in which they are embedded.

Under supportive conditions—including recognition of the caring role, access to reliable information, opportunities for emotional support, and guidance from professionals—young carers may develop functional competencies, such as organisational skills, health-related knowledge, and emotional sensitivity. In these contexts, caregiving can promote self-confidence and psychosocial growth. Conversely, in the absence of these conditions, caregiving responsibilities are often associated with persistent fatigue, emotional distress, social withdrawal, and reduced educational participation.

This distinction emphasises that caregiving tasks alone do not determine outcomes; instead, outcomes are mediated by factors such as professional awareness [54], timely support, and opportunities for respite and peer connection.

*Family-Level Findings.* The qualitative data show that, within families, care emerges as a dynamic and interdependent process embedded in daily life. The Whole Family Approach offers a valuable lens to interpret these interactions, highlighting that the well-being of each family member is interlinked [57,60]. Open communication, shared decision-making, and reciprocal emotional support can buffer the stresses associated with illness and caregiving. By contrast, high caregiving intensity with limited external assistance increases emotional strain and relational tensions, restricting the young carer’s social and academic engagement [21,63,64]. Tronto and Fisher’s Modes of Care [56] help illuminate these patterns by framing care as a continuum of moral and practical relations—caring about, taking care of, caregiving, and care-receiving. Families engaging in mutual, dialogical forms of care tend to maintain equilibrium and foster resilience, whereas families with limited recognition or support experience cumulative strain. This aligns with Metzing’s [49] conceptualisation of care as relational and evolving, in which roles and responsibilities require continuous negotiation across changing illness trajectories and developmental stages.

*Institutional-Level Findings.* Schools and healthcare providers are often the first to observe the consequences of young caring, yet their awareness and capacity to respond remain uneven. Institutional support depends on local resources, professional awareness, and cross-sector coordination [41]. Flexible attendance policies, carer-sensitive pedagogy, and integration of counselling services can mitigate educational disruption and promote continuity. However, the lack of coordination between education, health, and social care sectors continues to pose structural barriers [41].

Many professionals report a general willingness to support young carers: 55.8% indicated they could provide support, and 57.7% stated they could refer young carers to other services. However, only 44.7% reported familiarity with young carer terminology, and overall identification capacity reached 53.1%, with significant sectoral differences (χ^2^ (2, *N* = 2311) = 24.326, *p* < 0.001). Crucially, the ability to support was strongly associated with prior identification (χ^2^ (2, *N* = 2311) = 197.077, *p* < 0.001), demonstrating that recognition functions as a gateway to action.

At the same time, referral pathways differed significantly across sectors (χ^2^ (4, *N* = 2142) = 24.090, *p* < 0.001), indicating that navigation mechanisms and responsibilities are unevenly structured. These findings reveal a structural tension: professional willingness is present, yet awareness, identification, and coordination are inconsistently embedded within institutional arrangements. Support, therefore, remains contingent on individual initiative rather than guided by systematic procedures.

In line with the care arrangement perspective proposed by Nagl-Cupal et al. [51], professional awareness emerges as the critical interface between informal family care and formal service systems. However, the empirical evidence shows that this interface lacks structural stabilisation. Without formalised recognition processes, shared mandates, and coordinated cross-sector pathways, support cannot reliably move from episodic intervention to sustained continuity.

This structural gap between willingness and systemic embedding constitutes the central empirical rationale for the development of the *Caring in Context Framework*. The framework directly addresses the identified misalignment by integrating recognition, coordination, and responsibility across institutional and relational levels, thereby providing the structural architecture to translate awareness into durable support.

*Societal-Level Findings.* Broader cultural, political, and economic contexts shape how young carers are recognised and supported [28]. The empirical findings from both studies indicate that societal recognition of young carers in Switzerland remains structurally limited. In the nationwide survey, only 44.7% of professionals reported familiarity with relevant young carer terminology, and fewer than one-third perceived the issue as clearly relevant within their occupational context. These findings suggest that awareness has not yet been systematically anchored within professional education, policy discourse, or institutional mandates.

Sectoral differences in identification capacity and referral practices further point to the absence of a unified national framework, indicating that recognition depends heavily on local structures and individual engagement rather than on coherent policy guidance. Qualitative interviews reinforce this pattern of invisibility: families described limited public understanding of children’s caregiving roles, and young carers reported that their contributions were rarely acknowledged beyond the immediate family context.

Taken together, these findings suggest that young carers’ responsibilities remain insufficiently embedded within broader social and policy narratives. Their contributions are largely invisible within formal care statistics, service planning, and legislative discourse. The societal context, therefore, not only frames but also structurally conditions recognition, resource allocation, and support pathways. This highlights the need for policy-level anchoring and public visibility as integral components of a comprehensive support framework.

While legislation has advanced recognition of adult carers, young carers continue to be excluded from national policy frameworks. Cultural silence surrounding illness and mental health further contributes to underreporting and limited help-seeking. International evidence suggests that legal recognition, public awareness campaigns, and dedicated funding can strengthen social inclusion and well-being [9]. Recognising young carers as individuals with agency, rights, and competencies represents a paradigm shift necessary for equitable support.

*Integration: Leverage Points and Framework Domains.* Synthesising insights across these levels demonstrates that effective support for young carers requires cross-sectoral coordination, systemic alignment, and participatory approaches involving families, professionals, and policy stakeholders. As demonstrated by the Swiss data, awareness functions as the shared core element enabling identification, legitimisation, and coordination of support. The triangulated findings from this research study informed the development of the *Caring in Context Framework,* which identifies key leverage points for intervention—particularly during educational transitions or changes in caregiving intensity. Transitions, such as school changes or shifts in the care recipient’s health status, represent critical phases in which caring demands often intensify while support structures weaken. The framework advocates for integrated care pathways that combine individual counselling, family therapy, school-based support, and community outreach. Longitudinal and mixed-method evaluations are essential for assessing outcomes in terms of educational continuity, psychosocial well-being, and resilience.

Building on Metzing’s foundational work [49] and the empirical data, the framework distinguishes five interrelated domains of young carers’ support activities: (1) support for the care recipient, (2) household tasks, (3) tasks outside the household, (4) support for healthy family members, and (5) self-care and personal management. These domains encompass both the visible and invisible dimensions of care work and provide a practical structure for assessment and intervention. Addressing these domains through participatory approaches ensures that young carers and care recipients are actively involved in shaping support that reflects their lived realities. Incorporating these domains into professional practice ensures that the complex, relational, and developmental realities of young carers’ lives are acknowledged and supported.

*Synthesis and Implications.* In conclusion, this system framework moves beyond a deficit-oriented understanding of youth caregiving toward a strength-based, relational, and family-centred paradigm. By positioning awareness as the starting point rather than the endpoint of support, the *Caring in Context Framework* links recognition to participation, coordination, and continuity across the life course. It calls for coherent, sustainable strategies that recognise young carers as integral members of families and communities. Aligning policy, practice, and research through this lens offers a pathway to equitable and inclusive support structures that value both the caregiving contributions and the developmental needs of young carers.

*Methodological Considerations.* The *Caring in Context Framework* is derived from empirical data collected within the Swiss socio-political context, characterised by federalised health and education systems and regional variation in service provision. While this setting provides valuable insights into intersectoral fragmentation, transferability to more centralised welfare systems requires cautious interpretation.

The underlying studies relied on cross-sectional survey data and qualitative interviews. Consequently, causal relationships between awareness, coordination, and support outcomes cannot be established, and the findings reflect perspectives captured at a specific point in time. Self-report data may be influenced by recall bias, social desirability, or situational interpretations.

Finally, the *Caring in Context Framework* represents a theoretically informed synthesis of empirical findings rather than an intervention model that has been prospectively implemented and evaluated. The framework translates recurring empirical patterns—particularly those related to recognition, coordination, and family-centred practice—into a structured conceptual model. Its strength lies in linking relational care theory with multi-perspective empirical evidence across sectors. However, its practical applicability, scalability, and long-term impact require further validation through implementation research, longitudinal designs, and context-sensitive evaluation studies. Future research should therefore examine how the framework can be operationalised within institutional routines and how it influences measurable outcomes such as educational continuity, psychosocial well-being, and intersectoral coordination.

*Implications for Policy and Practice*. While further empirical validation of the proposed framework is required, the present findings already provide clear guidance for policy and practice. Policy frameworks should explicitly recognise young carers within national family, education, and health agendas, ensuring systematic access to counselling, financial assistance, and respite services. In this sense, the framework not only responds to empirical findings in the Swiss context but also aligns with broader international commitments to health equity, educational inclusion, and social participation. It translates global development principles into a structured, practice-oriented model applicable within welfare systems seeking sustainable and inclusive care arrangements. At the practice level, schools and healthcare institutions should implement structured screening and referral mechanisms, supported by professional training that promotes awareness and carer-sensitive practice. Interdisciplinary collaboration that links educators, healthcare professionals, and social services is critical for creating seamless support pathways. Rather than establishing additional service infrastructures, efforts should focus on the coordinated use of existing organisational structures. Such an approach enhances efficiency, strengthens coherence, and promotes long-term systemic sustainability [23].

Community organisations and youth programmes should be resourced to provide peer support and advocacy platforms that empower young carers to participate in shaping policies that affect their lives. Evidence from peer-support research reinforces this recommendation. Evaluations of structured group programmes show that peer support can reduce feelings of isolation, enhance emotional support, and strengthen coping strategies when facilitated in safe and professionally guided settings, with positive feedback reported by children, parents, and facilitators alike [46]. Guided young carer peer-support groups in other contexts further indicate gains in social inclusion, well-being, and life skills, while highlighting challenges related to accessibility and reach [45].

Taken together, embedding these measures within a family-centred, cross-sectoral, and sustainable framework aligns with international standards of inclusive care. It contributes to strengthening the visibility, resilience, and well-being of young carers and their families [19,23,43].

## 5. Conclusions

This research advances the international discourse on young carers by demonstrating that their situations cannot be adequately understood or addressed through isolated, child-focused, or task-oriented approaches. Instead, young carers’ experiences and outcomes must be situated within the broader contexts in which care takes place. By integrating conceptual clarification, nationwide quantitative evidence, and in-depth qualitative insights, the study shows that it is not caregiving per se, but the surrounding conditions of care—particularly recognition, coordination, and family-centred support across systems—that decisively shape young carers’ developmental trajectories.

The central contribution of this research is the *Caring in Context Framework*—a family-centred, and cross-sectoral framework designed to support young carers. It consolidates prior insights while moving beyond isolated interventions by linking individual, family, institutional, and societal levels. By integrating ethical, relational, and systemic dimensions of care, it bridges lived experiences, professional practice, and institutional responsibilities to enable coordinated prevention, mitigation, and support across settings.

The framework captures the breadth of family caregiving through a set of interconnected support domains, recognising young carers as active contributors and emphasising that their well-being depends on supportive, coordinated, family-sensitive conditions rather than individual resilience.

A key strength of the *Caring in Context Framework* lies in making structurally visible what often remains obscured: young carers emerge at the intersection of fragmented education, health, and social systems. The findings show that without systematic recognition, coordinated pathways, and family-centred engagement, support remains episodic—regardless of professional goodwill.

The framework’s central innovation is its explicit coordination function. Rather than proposing new specialised services, it emphasises the strategic alignment of existing structures, the clarification of responsibilities, and cross-sector integration. In welfare contexts facing increasing financial pressure, this shift from service expansion to systemic coordination offers a pragmatic and sustainable policy direction.

By embedding recognition procedures and shared mandates within current institutional arrangements, the framework translates awareness into continuity. Although empirically grounded in the Swiss context, its multi-level architecture and adaptable coordination logic make it transferable to other welfare systems seeking coherent, cost-conscious, and inclusive support strategies.

From a policy and practice perspective, *Caring in Context* offers a coherent foundation for action. It highlights the urgency of embedding young carers systematically within national education, health, and social policies; establishing formal mechanisms for intersectoral coordination; and investing in professional training that enables early identification and sustained support. Beyond structural reforms, the framework also underscores the importance of public recognition and destigmatisation as essential enabling conditions for lasting change.

In conclusion, recognising and supporting young carers is not merely a matter of individual welfare but a structural and ethical obligation. The *Caring in Context Framework* strengthens existing approaches by integrating ethical care theory with empirical evidence and system-level analysis. It provides a transferable, family-centred, and cross-sectoral foundation for developing sustainable support systems that safeguard young carers’ rights to education, health, and social participation—while acknowledging their essential contributions to family care.

## Data Availability

No new data were created or analysed in this study. Data sharing is not applicable to this article.

## Abbreviations

The following abbreviations are used in this manuscript:

SDG	Sustainable Development Goals

## Appendix A

Table A1 provides an overview of the two empirical studies forming the foundation of the *Caring in Context Framework*. Study 1 examined institutional awareness and support practices among professionals, while Study 2 explored the lived experiences of young carers and their families. Together, the studies informed the identification of recognition, coordination, and family-centred engagement as central structural mechanisms underlying effective support.

**Table A1:** Overview of Empirical Studies Underpinning the Caring in Context Framework.

Category	Study 1: Professional Awareness and Support Practices	Study 2: Lived Experiences of Young Carers and Families
Study Period	2016–2018	2018–2020
Primary Aim	To examine awareness, identification, perceived responsibility, and support practices among professionals in education, healthcare, and social services	To explore how young carers, young adult carers, and care recipients experience caregiving, professional involvement, and support in everyday life
Design	Cross-sectional quantitative online survey	Qualitative semi-structured interview study
Participants (Final Sample)	*N* = 2142 professionals (education, healthcare, social services)	*N* = 20 (young carers, young adult carers, care recipients)
Sampling Strategy	Purposive and snowball sampling via national institutional contacts	Purposive sampling via professional networks, support services, and prior research contacts
Recruitment Procedure	Email invitations to institutions and professionals; nationwide outreach; three-language survey (German, French, Italian)	Individual invitations; voluntary participation; informed consent; interviews conducted face-to-face
Data Collection Method	Standardised questionnaire including closed-ended and open-ended items	Audio-recorded semi-structured interviews; verbatim transcription
Key Variables/Focus Areas	Familiarity with young carer terminology; perceived relevance; identification capacity; ability to support; referral practices; sectoral differences	Care burden; access to information; role conflict; relational dynamics; professional engagement; continuity of support
Data Analysis	Descriptive statistics (frequencies, percentages, 95% CI); Chi-square tests; effect sizes (Phi); significance level α = 0.05; IBM SPSS	Qualitative content analysis; inductive and deductive coding; iterative team-based analysis
Central Empirical Findings	Limited familiarity; identification capacity; significant sector differences; strong association between identification and support; fragmented referral structures	Structural invisibility of young carers; information gaps; role conflict; ambivalence in families; fragmented coordination and discontinuity of care
Level of Analysis	Institutional/professional level	Individual and family level
Contribution to Framework Development	Identified recognition and coordination as structural mechanisms shaping institutional responses	Demonstrated how systemic gaps translate into lived burden, role expansion, and family-level compensation mechanisms
Publications	Leu, A.; Wepf, H.; Sempik, J.; Nagl-Cupal, M.; Becker, S.; Jung, C.; Frech, M. Caring in Mind? Professionals’ Awareness of Young Carers and Young Adult Carers in Switzerland [30]. Frech, M.; Wepf, H.; Nagl-Cupal, M.; Becker, S.; Leu, A. Ready and Able? Professional Awareness and Responses to Young Carers in Switzerland [54].	Frech, M.; Berger, F.; Rabhi-Sidler, S.; Nagl-Cupal, M.; Becker, S.; Leu, A. How Professional Support for Young Carers Benefits from a Salutogenic Approach [53].
